# Schedule-dependent antitumor effects of 5-fluorouracil combined with sorafenib in hepatocellular carcinoma

**DOI:** 10.1186/1471-2407-13-363

**Published:** 2013-07-29

**Authors:** Lifen Deng, Zhenggang Ren, Qingan Jia, Weizhong Wu, Hujia Shen, Yanhong Wang

**Affiliations:** 1 Liver Cancer Institute, Zhongshan Hospital, Fudan University, Shanghai, 200032, People’s Republic of China; 2 Key Laboratory of Carcinogenesis and Cancer Invasion (Fudan University), Ministry of Education, Shanghai, 200032, People’s Republic of China

**Keywords:** Hepatocellular carcinoma, Sorafenib, 5-fluorouracil, Cell cycle arrest

## Abstract

**Background:**

Recently, a phase II clinical trial in hepatocellular carcinoma (HCC) has suggested that the combination of sorafenib and 5-fluorouracil (5-FU) is feasible and side effects are manageable. However, preclinical experimental data explaining the interaction mechanism(s) are lacking. Our objective is to investigate the anticancer efficacy and mechanism of combined sorafenib and 5-FU therapy in vitro in HCC cell lines MHCC97H and SMMC-7721.

**Methods:**

Drug effects on cell proliferation were evaluated by cell viability assays. Combined-effects analyses were conducted according to the median-effect principle. Cell cycle distribution was measured by flow cytometry. Expression levels of proteins related to the RAF/MEK/ERK and STAT3 pathways and to cell cycle progression (cyclin D1) were determined by western blot analysis.

**Results:**

Sorafenib and 5-FU alone or in combination showed significant efficacy in inhibiting cell proliferation in both cell lines tested. However, a schedule-dependent combined effect, associated with the order of compound treatments, was observed. Efficacy was synergistic with 5-FU pretreatment followed by sorafenib, but it was antagonistic with the reverse treatment order. Sorafenib pretreatment resulted in a significant increase in the half inhibitory concentration (IC50) of 5-FU in both cell lines. Sorafenib induced G1-phase arrest and significantly decreased the proportion of cells in S phase when administrated alone or followed by 5-FU. The RAF/MEK/ERK and STAT3 pathways were blocked and cyclin D1 expression was down regulated significantly in both cell lines by sorafenib; whereas, the kinase pathways were hardly affected by 5-FU, and cyclin D1 expression was up regulated.

**Conclusions:**

Antitumor activity of sorafenib and 5-FU, alone or in combination, is seen in HCC cell lines. The nature of the combined effects, however, depends on the particular cell line and treatment order of the two compounds. Sorafenib appears to reduce sensitivity to 5-FU through down regulation of cyclin D1 expression by inhibiting RAF/MEK/ERK and STAT3 signaling, resulting in G1-phase arrest and reduction of the S-phase cell subpopulation when 5-FU is administrated after sorafenib, in which situation, combination treatment of the two agents results in antagonism; on the other hand, when sorafenib is administrated afterward, it can continue to work since it is not cell cycle specific, as a result, combination treatment of the two agents shows an additive-to-synergistic effect.

## Background

Hepatocellular carcinoma (HCC) is the sixth most common malignancy worldwide and ranks as the third leading cause of cancer-related death, accounting for 748,300 new cases and 695,900 deaths worldwide per year. Half of these cases and deaths are estimated to occur in China [1]. However, only approximately 30%–40% of patients are diagnosed in an early stage (0 or A) according to the Barcelona Clinic Liver Cancer staging system [2], which defines patients who are suitable for potentially curative approaches, such as surgical therapies (resection and liver transplantation) and locoregional procedures (radiofrequency ablation). For patients who meet the criteria for the intermediate stage (multinodular HCC, relatively preserved liver function, absence of cancer-related symptoms, and no evidence of vascular invasion or extrahepatic spread), transcatheter arterial chemoembolization (TACE) has been established as the standard of care, and this treatment may achieve a partial response or complete necrosis [3]. For patients with advanced HCC, sorafenib is the first agent discovered to result in favorable overall survival [4]. Regional hepatic arterial infusion chemotherapy (HAIC) has also been used in patients with advanced HCC in cases in which TACE is not indicated or is ineffective [5,6].

The technique of TACE, including which drug is administrated, the scheduled followed after the first TACE or the follow-up imaging modalities, varies worldwide with no clear consensus. Among the agents commonly used in TACE and HAIC to inhibit cancer cell growth, 5-Fluorouracil (5-FU) is a widely used chemotherapeutic drug. It initiates apoptosis by targeting thymidylate synthase (TS) and direct incorporation of 5-FU metabolites into DNA and RNA. However, its efficacy in HCC is poor [7], and the compound is associated with acquired and intrinsic resistance.

Sorafenib (BAY 43-9006, Nexavar) is an oral multikinase inhibitor that inhibits the serine-threonine kinases C-Raf and B-Raf, the receptor tyrosine kinase activity of vascular endothelial growth factor receptors -1, -2, and -3, platelet-derived growth factor receptor β, the receptor for the macrophage-colony stimulating factor (FLT3), Ret, and c-Kit. These kinases are involved in cell proliferation and tumor angiogenesis [8,9]. In addition, increasingly more studies have pointed out that signal transducer and activator of transcription 3 (STAT3) is a major kinase-independent target of sorafenib in HCC [10,11].

Recently, a phase II clinical trial has suggested that the combination of sorafenib and 5-fluorouracil is feasible, and the side effects are manageable for patients carefully selected for liver function and performance status [12]. However, preclinical experimental data explaining interaction mechanisms are widely missing. One previous study in our institute found that resistance to 5-FU was significantly associated with basal p-ERK expression levels in HCC cell lines while sorafenib inhibited ERK phosphorylation in a dose-dependent manner [13]. Chances are combination of sorafenib and 5-FU would exert a synergetic effect with the hypothesis that sorafenib could reverse the resistance to 5-FU of HCC cells by inhibiting p-ERK expressions. However, it is known that 5-FU is an S-phase-specific agent, whereas sorafenib causes G1-phase arrest in tumor cells [14]. The latter implies that sorafenib treatment would decrease the proportion of cells in S phase. And in such situation, tumor cells might become less susceptible to the 5-FU action. Therefore, the effects of combined sorafenib and 5-FU co-administration are uncertain.

In the present study, we initiated an in vitro study in HCC cell lines MHCC 97H and SMMC-7721 to investigate the anticancer efficacy and molecular mechanisms of combined administration of sorafenib and 5-FU.

## Methods

### Drug preparations

Sorafenib (Nexavar), N-(3-trifluoromethyl-4-chlorophenyl)-N-(4-(2-methylcarbamoylyridin-4-yl)oxy-phenyl) urea, was purchased from BioVision, Inc. (Milpitas, CA, USA). The compound was dissolved in 100% dimethyl sulfoxide (DMSO; Sigma-Aldrich, St Louis, MO, USA) and diluted with Dulbecco's modified Eagle's medium (DMEM) or RPMI 1640 to the desired concentration; a final DMSO concentration of 0.1% (v/v) was present in cell studies. As solvent control, 0.1% DMSO alone was added to cultures. 5-Fluorouracil injection was purchased from Shanghai Xudong Haipu Pharmaceutical Co, Ltd. (Shanghai, China) and was diluted directly with cell culture medium to the desired concentration.

### Cell lines

Human HCC tumor cell lines MHCC97H and SMMC-7721 were obtained from the Liver Cancer Institute of Fudan University (Shanghai, China) and cultured in DMEM or RPMI 1640 containing 10% v/v fetal bovine serum at 37°C in a humidified incubator containing 5% CO_2_. Unless otherwise indicated, cell culture reagents were purchased from GIBCO BRL (Grand Island, NE, USA).

### Cell viability assay

Cells were plated in 96-well microtiter plates (4,000 per well) in 100 μL of serum-containing medium and incubated overnight at 37°C in the culture incubator. On the following day, the medium was replaced with fresh medium containing sorafenib, 5-FU, or a combination of the two agents at various concentrations. Treatment with sorafenib was done for 24 h at concentrations of 0, 0.25, 0.5, 1, 4, 8, 16, 32, 64, or 128 μM; that with 5-FU was for 48 h at concentrations of 0, 0.1, 1, 2, 4, 8, 16, 32, 64, 128, or 256 mg/L. Cell viability was measured using the Cell Counting Kit-8 (Dojindo Laboratories, Kumamoto, Japan) according to the manufacturer's instructions. The half maximal inhibitory concentration (IC50) values were calculated by nonlinear regression analysis using GraphPad Prism version 5.0 software (GraphPad Software, Inc., San Diego, CA, USA).

Combination index (CI) values were calculated using the median effect analysis method. A synergistic effect is defined as CI < 1, an additive effect as CI = 1, and an antagonistic effect as CI > 1.

Each condition was tested six times, and the results were confirmed in at least three independent experiments.

To further investigate combined effects of sorafenib and 5-FU on cell proliferation, growth inhibition, cell cycle distribution and pathways activities, six treatment groups were designed as follows: group control (0.1% DMSO); group S (treatment with 8 μM sorafenib for 24 h); group F (treatment with 4 mg/L 5-FU for 48 h); group (S + F) (concurrent treatment with 8 μM sorafenib and 4 mg/L 5-FU for 48 h); group S + F (8 μM sorafenib pretreatment for 24 h followed by 4 mg/L 5-FU treatment for another 48 h); group F + S (4 mg/L 5-FU treatment followed by 8 μM sorafenib for another 24 h).

### Cell cycle assays

Exponentially growing cells were starved in serum-free medium for 24 h, after which they were grown in medium containing 10% serum with the compounds 8 μM sorafenib for 24 h or 4 mg/L 5-FU for 48 h, either alone or in combination patterns. Cell cycle analyses and quantification of genomic DNA fragmentation were performed using the Cell Cycle Detection Kit (KeyGEN, Nanjing, China) according to the manufacturer’s protocol. Cell cycle distributions were analyzed by flow cytometry with a Becton Dickinson FACS Calibur.

### Western blot analysis

To prepare whole-cell protein extracts, cells were washed twice with phosphate-buffered saline and then lysed with a modified radio-immunoprecipitation assay buffer (50 mM Tris–HCl pH 7.4, 1% v/v NP-40, 0.25% v/v sodium deoxycholate, 150 mM NaCl, 1 mM EDTA, 1 mM PMSF, 1 mg/mL of protease inhibitors (leupeptin and pepstatin), 1 mM Na_3_VO_4_, and 1 mM NaF) on ice for 30 min. Insoluble material was removed by centrifugation at 12,000 p/min for 15 min at 4°C. The protein concentration of cell lysates was measured using the Bradford Protein Assay Kit (Beyotime, Shanghai, China), and 30 μg of protein samples were loaded on 10% polyacrylamide gels containing sodium dodecyl sulfate and separated by electrophoresis at a constant voltage of 70 V for 2 h and transferred onto 0.45-μm polyvinylidene fluoride membranes (Millipore Corporation, Billerica, MA, USA) at a constant voltage of 100 V for 3 h at 0°C. The membranes were probed with the specific primary antibodies followed by a horseradish peroxidase-conjugate secondary antibody (1:5,000) and detected by enhanced chemiluminescence (ECL kit from Pierce, Rockford, IL, USA). The following primary antibodies were used: anti-C-RAF (1:1,000), anti-phospho-C-RAF (1:1,000), anti-ERK1/2 (1:1,000), and anti-phospho-ERK1/2 (Thr202/Tyr204) (1:1,000) from Cell Signaling Technology, Inc. (Danvers, MA, USA); anti-STAT-3 (1:1,000) and anti-phospho-STAT-3 (Tyr705) (1:1,000) from Abcam (Cambridge, MA, USA); and anti-cyclin D1 (1:1000) and anti-β-actin from Beyotime. Unless otherwise indicated, immunoblot reagents were purchased from Beyotime.

### Statistical analysis

Statistical analysis was performed with SPSS 17.0 software (SPSS, Chicago, IL, USA). Measured values are expressed as mean ± standard deviation. Analysis of variance and least significant difference were used to evaluate statistical significance of differences between groups, and a P value of <0.05 was considered statistically significant.

## Results

### Antitumor effects of sorafenib and 5-FU in HCC cell lines

**Figure 1:**
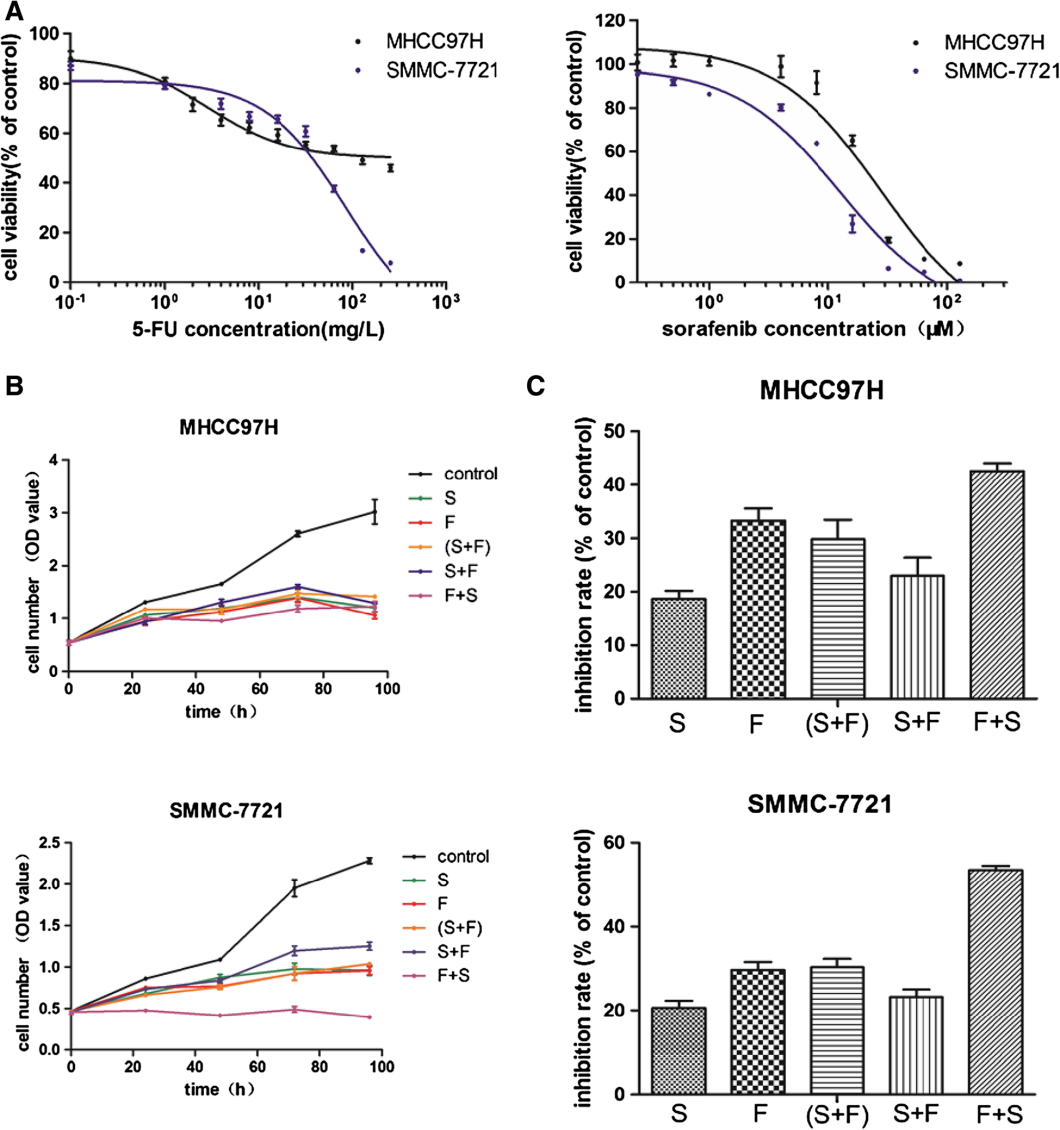
**Antitumor effects of sorafenib and 5-fluorouracil (5-FU), alone or in combination, in hepatocellular carcinoma (HCC) cell lines. (A)** Dose–response curves, correlating drug dose and cell viability, depict the effects of sorafenib and 5-FU on different HCC cell lines. The cell survival fraction is expressed relative to the untreated cells, set at 100. **(B)** Antiproliferation effects of 5-FU (4 mg/L, 48 h) and sorafenib (8 μM, 24 h), alone or in combination in different treatment sequences, in HCC cell lines. The cell numbers are represented as optical density (OD) values. **(C)** Inhibition rates of 5-FU (4 mg/L, 48 h) or sorafenib (8 μM, 24 h), alone or in combination in different treatment sequences, in HCC cell lines. The cell inhibition fraction is expressed relative to the untreated cells, set at 100. In this figure, values represent the mean ± standard deviation (SD), and each is the average of three independent determinations, with six replicates per experiment. S, sorafenib; F, 5-FU.

Sorafenib and 5-FU both inhibited cell proliferation of the two HCC cell lines in a dose-dependent manner. The IC50 values of sorafenib were 17.82 ± 2.04 μM and 15.52 ± 0.95 μM in MHCC97H and SMMC-7721 cells, respectively, and the corresponding IC50 values of 5-FU were 116.59 ± 62.04 mg/L and 47.19 ± 13.02 mg/L, respectively. The dose–response curves for the two HCC cell lines are shown in Figure 1(A).

To evaluate the combined effects of sorafenib and 5-FU on cell proliferation and growth inhibition, six treatment groups were designed as in section “Methods”. The cell proliferation conditions of the six groups are shown in Figure 1(B), and inhibition rates of the six groups are listed in Figure 1(C) and Table 1. Our results generally suggest that inhibitory effects were equipotent to 5-FU monotherapy when 5-FU was concurrently administrated with sorafenib, better in the 5-FU-pretreated sequence, and, conversely, worse in the sorafenib pretreatment schedule (P values are shown in Table 1). That is, sequential treatment using 5-FU followed by sorafenib seems to be the optimal schedule for combined administration of the two agents.

**Table 1:** Inhibition rates (% of control) of sorafenib and 5-fluorouracil (5-FU), alone or in combination, in hepatocellular carcinoma (HCC) cells

**Cell line**	**S**	**F**	**(S + F)/ *p *-value(vs. F)**	**S + F/ *p *-value(vs. F)**	**F + S/ *p *-value(vs. F)**
MHCC97H	18.63 ± 3.82	33.30 ± 5.67	29.87 ± 8.83/*p* = 0.328	22.98 ± 5.93/*p* = 0.023	42.57 ± 3.29/*p* = 0.017
SMMC-7721	20.60 ± 3.83	29.65 ± 4.74	30.35 ± 4.86/*p* = 0.781	23.23 ± 4.43/*p* = 0.016	53.50 ± 1.97/*p* = 0.000

*S:* sorafenib, F: 5-FU.

To further explore whether the combination of sorafenib with 5-FU results in synergism, additivity, or antagonism of inhibition of cell proliferation, combination index (CI) values were calculated using the median effect analysis method [15]. Sorafenib and 5-FU were administrated at certain concentration ratios in different sequences. The CI values are summarized in Table 2. Our data indicate that combination treatment of sorafenib and 5-FU largely resulted in antagonism in MHCC97H cells regardless of treatment order, with a degressive trend as drug concentrations increase. Further analysis indicated that the CI values of the 5-FU-pretreated group were smaller than those of the sorafenib-pretreated group and drew near 1 as drug concentrations increased, which indicated an additive-to-synergistic effect. Situations in SMMC-7721 cells were similar except that pretreatment with 5-FU showed an apparent synergistic effect.

**Table 2:** Combination index (CI) values of sorafenib and 5-fluorouracil (5-FU) combination in different treatment sequences in hepatocellular carcinoma (HCC) cells

**Sorafenib concentration (μM)**	**5-FU concentration (mg/L)**	**MHCC97H**			**SMMC-7721**		
		**(S + F)**	**S + F**	**F + S**	**(S + F)**	**S + F**	**F + S**
1	0.5	4.33 ± 1.73	32.84 ± 4.26	3.88 ± 1.73	112.45 ± 28.23	67.40 ± 15.67	13.63 ± 9.81
2	1	2.07 ± 0.44	9.70 ± 3.96	5.20 ± 2.17	1.95 ± 1.01	13.50 ± 9.43	0.50 ± 0.34
4	2	1.47 ± 0.25	3.69 ± 2.08	3.25 ± 2.57	1.33 ± 0.38	1.80 ± 0.11	0.41 ± 0.20
8	4	1.18 ± 0.28	2.33 ± 1.24	1.13 ± 0.27	1.45 ± 0.33	1.66 ± 0.17	0.38 ± 0.05
16	8	0.99 ± 0.40	1.26 ± 0.30	0.75 ± 0.15	1.02 ± 0.09	1.71 ± 0.06	0.40 ± 0.04

*CI* Combination index. CI < 1 indicate synergy between the drugs; CI = 1 indicates additivity; and CI > 1 indicates antagonism. Values are expressed as the mean ± SD of at least three independent experiments performed in triplicate. *S:* sorafenib, *F*: 5-FU.

### Sensitivity of HCC cells to 5-FU in combination with sorafenib

**Figure 2:**
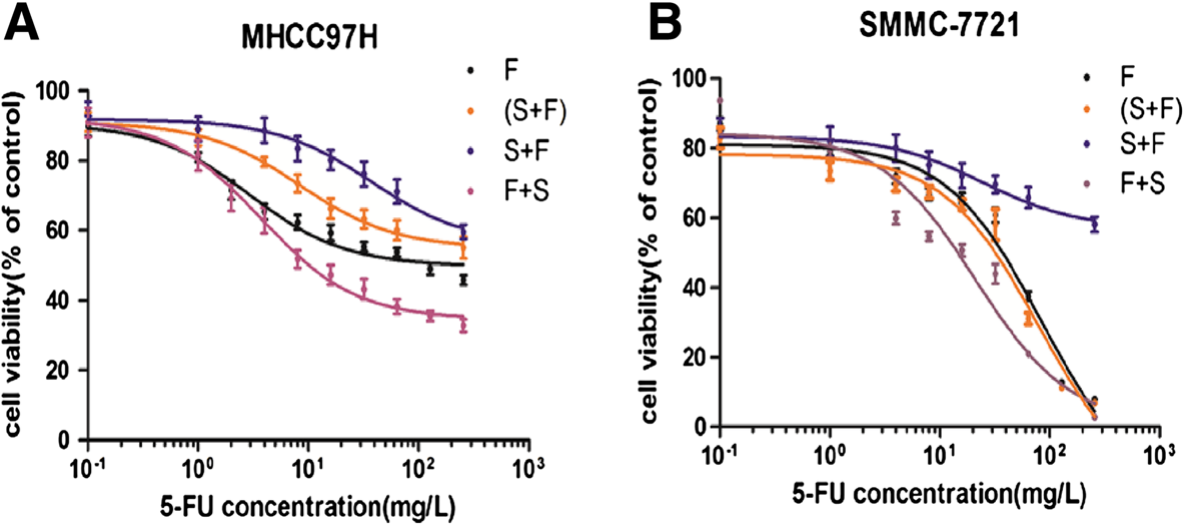
**Sensitivity of hepatocellular carcinoma (HCC) cells to 5-fluorouracil (5-FU) when treated in combination with sorafenib in vitro.** Dose–response curves of 5-FU alone and combined with sorafenib, in different treatment sequences, are shown. HCC cell lines MHCC97H **(A)** and SMCC-7721 **(B)** were exposed to escalating doses of 5-FU, alone or combined with 8 μM sorafenib, in different treatment sequences. The cell survival fraction is expressed relative to the untreated cells, set at 100, and is expressed as the mean ± SD. Each value represents the average of three independent determinations with six replicates per experiment. Bars indicate standard error. S, sorafeinb; F, 5-FU.

The sensitivity of HCC cell lines to 5-FU was determined by calculating the IC50 values from results of cell viability assays. In these experiments, four treatment groups were tested: group F (single treatment with 5-FU); group (S + F) (concurrent treatment with 5-FU and 8 μM sorafenib); group S + F (8 μM sorafenib pretreament for 24 h followed by 5-FU treatment); and group F + S (5-FU pretreatment followed by 8 μM sorafenib for another 24 h). Dose–response curves are shown in Figure 2, and IC50 values for 5-FU treatment of the four groups are listed in Table 3. Sensitivity to 5-FU varied greatly, depending on compound treatment order: sorafenib dramatically decreased the sensitivity to 5-FU when it was administrated prior to 5-FU, with the IC50 values increasing significantly (P < 0.001 for both) in both MHCC97H and SMMC-7721 cells. Conversely, the IC50 values of 5-FU decreased in both cell lines when sorafenib was administrated afterward.

**Table 3:** **Sensitivity of hepatocellular carcinoma (HCC) cells to 5-fluorouracil (5-FU) (IC**_**50 **_**of 5-FU (mg/L)) in different treatment strategies**

**Cell line**	**F**	**(S + F)/ *p *-value (vs. F)**	**S + F/ *p *-value (vs. F)**	**F + S/ *p *-value (vs. F)**
MHCC97H	116.59 ± 62.04	271.63 ± 57.08/*p* = 0.002	477.46 ± 146.45/*p* = 0.000	25.45 ± 9.72/*p* = 0.042
SMMC-7721	47.19 ± 13.02	43.16 ± 8.76/*p* = 0.948	1371.26 ± 237.70/*p* = 0.000	9.47 ± 1.03/*p* = 0.568

*S:* sorafenib, *F*: 5-FU.

### Effects of sorafenib and 5-FU on cell cycle progress in HCC cell lines

**Figure 3:**
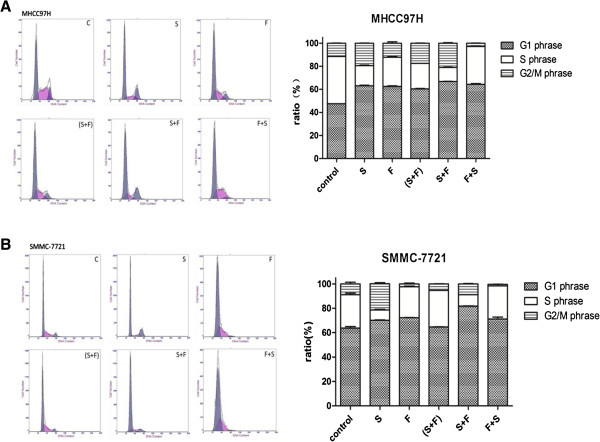
**Effects of sorafenib and 5-fluorouracil (5-FU) on cell cycle progression in the hepatocellular carcinoma (HCC) cell lines MHCC97H (A) and SMMC-7721 (B).** After serum starvation for 24 h, cells were exposed to sorafenib (8 μM, 24 h), 5-FU (4 mg/L, 48 h), or combination treatments of the two agents. The cell cycle distributions were then analyzed by flow cytometry. C, control; S, sorafeinb; F, 5-FU.

Six treatment groups (group control, S, F, (S + F), S + F, and F + S, as described above) were tested. Cell cycle distributions are shown in Figure 3 and Tables 4 and 5. Our data indicate that sorafenib induced a G1-cell cycle arrest and significantly decreased the proportion of cells in S phase in both HCC cell lines when it was administrated alone or followed by 5-FU: proportions of cells in G1 phase increased from 47.53 ± 0.06% to 63.03 ± 0.95% and 66.70 ± 0.30% (P < 0.001 for both) in the two groups respectively and proportions of cells in S phase decreased from 40.97 ± 0.15% to 17.43 ± 0.85% and 12.27 ± 0.45% (P < 0.001 for both) in MHCC97H cells. For SMMC-7721 cells, proportions of cells in G1 phase increased from 63.83 ± 1.94% to 70.07 ± 0.70% and 81.83 ± 0.35% respectively (P < 0.001 for both) and proportions of cells in S phase decreased from 27.17 ± 2.41% to 8.45 ± 1.03% and 9.23 ± 0.12% respectively (P < 0.001 for both). Simultaneous treatment or pretreatment with 5-FU reversed this effect to some extent.

**Table 4:** Cell cycle distribution of MHCC97H cells after different drug treatments

	**G1 phase (%)/ *p *-value (vs. control)**	**S phase (%)/ *p *-value (vs. control)**	**G2/M phase (%)/ *p *-value (vs. control)**
Control	47.53 ± 0.06	40.97 ± 0.15	11.50 ± 0.20
S	63.03 ± 0.95/*p* = 0.000	17.43 ± 0.85/*p* = 0.000	19.50 ± 0.10/*p* = 0.000
F	62.60 ± 0.70/*p* = 0.000	25.23 ± 0.72/*p* = 0.000	12.17 ± 1.95/*p* = 0.367
(S + F)	60.50 ± 0.50/*p* = 0.000	22.00 ± 0.10/*p* = 0.000	17.47 ± 0.35/*p* = 0.000
S + F	66.70 ± 0.30/*p* = 0.000	12.27 ± 0.45/*p* = 0.000	21.03 ± 0.75/*p* = 0.000
F + S	64.30 ± 1.10/*p* = 0.000	32.80 ± 1.00/*p* = 0.000	2.91 ± 0.07/*p* = 0.000

*S:* sorafenib, *F*: 5-FU.

**Table 5:** Cell cycle distribution of SMMC-7721 cells after different drug treatments

	**G1 phase (%)/ *p *-value (vs control)**	**S phase (%)/ *p *-value (vs control)**	**G2/M phase (%)/ *p *-value (vs control)**
Control	63.83 ± 1.94	27.17 ± 2.41	9.00 ± 2.26
S	70.07 ± 0.70/*p* = 0.000	8.45 ± 1.03/*p* = 0.000	21.51 ± 1.63/*p* = 0.000
F	72.23 ± 0.35/*p* = 0.000	25.50 ± 0.80/*p* = 0.188	2.27 ± 1.15/*p* = 0.000
(S + F)	64.73 ± 0.15/*p* = 0.458	29.90 ± 0.10/*p* = 0.041	5.37 ± 0.27/*p* = 0.004
S + F	81.83 ± 0.35/*p* = 0.000	9.23 ± 0.12/*p* = 0.000	8.92 ± 0.44/*p* = 0.939
F + S	71.22 ± 2.80/*p* = 0.000	27.20 ± 2.30/*p* = 0.978	1.08 ± 0.40/*p* = 0.000

*S:* sorafenib, *F*: 5-FU.

### Activation of RAF/MEK/ERK and STAT3 pathways and expression of cyclin D1

**Figure 4:**
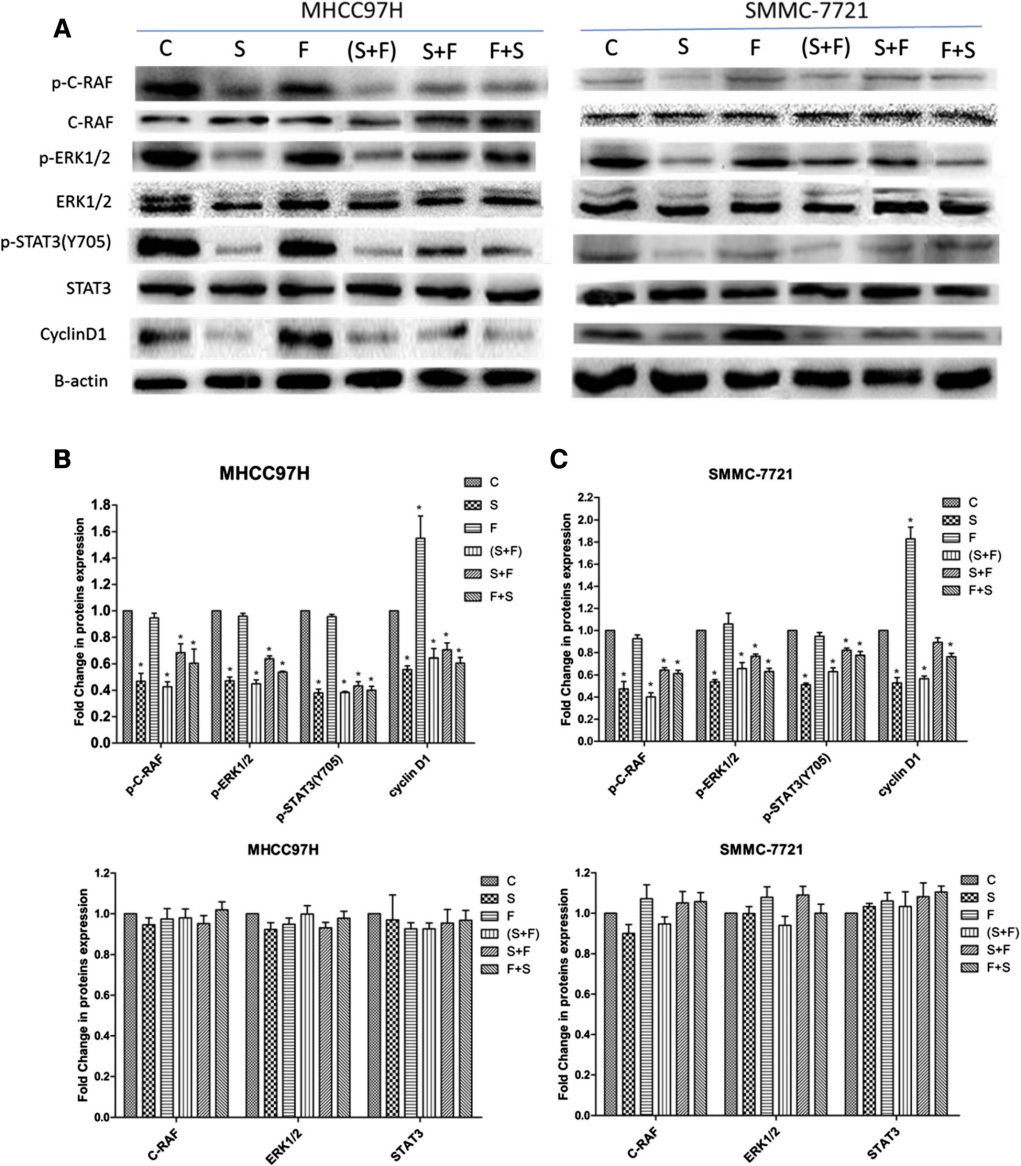
**Activation of RAF/MEK/ERK and STAT3 pathways and expression levels of cyclin D1 after treatment with sorafenib and 5-fluorouracil (5-FU).** Cells were treated with sorafenib (8 μM, 24 h), 5-FU (4 mg/L, 48 h), or combination of the two agents in different treatment sequences. Western blot analysis was performed to detect the expression levels of p-C-RAF, total C-RAF, p-ERK1/2, total ERK1/2, p-STAT3 (Y705), total STAT3, and cyclin D1. Loading controls were carried out by probing the blots for β-actin. Representative western blots **(A)** and quantification analysis **(B** and **C)** are shown. Asterisks (*) indicate significant differences in protein expression (P < 0.05). C, control; S, sorafeinb; F, 5-FU.

To identify the molecule mechanism of interactions between sorafenib and 5-FU, expression levels of proteins related to RAF/MEK/ERK and STAT3 pathways and to cell cycle progression (cyclin D1) were measured. Results showed that the levels of phosphorylated C-RAF, ERK, and STAT3 were significantly down regulated after sorafenib treatment in both cell lines (P < 0.001). Similar results were observed when sorafenib was concurrently administrated with 5-FU. Sequential therapies as well showed down-regulatory effects on expression of these proteins, although the differences were less than seen with sorafenib monotherapy. These pathways remained unchanged after exposure to 5-FU monotherapy. Moreover, sorafenib significantly down regulated cyclin D1 expression (P < 0.001), while 5-FU played an opposite role in both cell lines. Combined treatments also induced cyclin D1 down regulation, although the differences were less significant (Figure 4, Tables 6 and 7).

**Table 6:** Relative expression levels of proteins in MHCC97H cells after different drug treatments

	**C**	**S/ *p *-value (vs. control)**	**F/ *p *-value (vs. control)**	**(S + F)/ *p *-value (vs. control)**	**S + F/ *p *-value (vs. control)**	**F + S/ *p *-value (vs. control)**
p-C-RAF	1	0.47 ± 0.10/*p* = 0.000	0.95 ± 0.06/*p* = 0.544	0.43 ± 0.07/*p* = 0.000	0.68 ± 0.12/*p* = 0.003	0.61 ± 0.18/*p* = 0.001
C-RAF	1	0.95 ± 0.06/*p* = 0.336	0.97 ± 0.09/*p* = 0.631	0.98 ± 0.08/*p* = 0.703	0.95 ± 0.07/*p* = 0.386	1.02 ± 0.07/*p* = 0.737
p-ERK1/2	1	0.47 ± 0.05/*p* = 0.000	0.96 ± 0.04/*p* = 0.200	0.45 ± 0.05/*p* = 0.000	0.64 ± 0.04/*p* = 0.000	0.54 ± 0.01/*p* = 0.000
ERK1/2	1	0.92 ± 0.06/*p* = 0.099	0.95 ± 0.05/*p* = 0.255	1.00 ± 0.07/*p* = 0.965	0.93 ± 0.05/*p* = 0.133	0.98 ± 0.06/*p* = 0.611
p-STAT3(Y705)	1	0.38 ± 0.05/*p* = 0.000	0.96 ± 0.03/*p* = 0.193	0.38 ± 0.02/*p* = 0.000	0.43 ± 0.06/*p* = 0.000	0.40 ± 0.05/*p* = 0.000
STAT3	1	0.97 ± 0.21/*p* = 0.734	0.92 ± 0.05/*p* = 0.416	0.93 ± 0.05/*p* = 0.417	0.95 ± 0.12/*p* = 0.612	0.97 ± 0.08/*p* = 0.720
Cyclin D1	1	0.56 ± 0.05/*p* = 0.002	1.55 ± 0.29/*p* = 0.000	0.64 ± 0.12/*p* = 0.008	0.70 ± 0.09/*p* = 0.023	0.61 ± 0.07/*p* = 0.004

*C:* control, *S:* sorafenib, *F*: 5-FU.

**Table 7:** Relative expression levels of proteins in SMMC-7721 cells after different drug treatments

	**C**	**S/ *p *-value (vs. control)**	**F/ *p *-value (vs. control)**	**(S + F)/ *p *-value (vs. control)**	**S + F/ *p *-value (vs. control)**	**F + S/ *p *-value (vs. control)**
p-C-RAF	1	0.47 ± 0.12/*p* = 0.000	0.93 ± 0.06/*p* = 0.185	0.40 ± 0.07/*p* = 0.000	0.64 ± 0.04/*p* = 0.000	0.61 ± 0.05/*p* = 0.000
C-RAF	1	0.90 ± 0.08/*p* = 0.154	1.07 ± 0.12/*p* = 0.302	0.95 ± 0.06/*p* = 0.431	1.05 ± 0.10/*p* = 0.459	1.06 ± 0.08/*p* = 0.408
p-ERK1/2	1	0.54 ± 0.03/*p* = 0.000	1.06 ± 0.12/*p* = 0.403	0.66 ± 0.09/*p* = 0.000	0.77 ± 0.03/*p* = 0.005	0.63 ± 0.05/*p* = 0.000
ERK1/2	1	1.00 ± 0.06/*p* = 0.971	1.08 ± 0.09/*p* = 0.193	0.94 ± 0.07/*p* = 0.307	1.09 ± 0.07/*p* = 0.141	1.00 ± 0.08/*p* = 0.996
p-STAT3(Y705)	1	0.51 ± 0.02/*p* = 0.000	0.95 ± 0.06/*p* = 0.180	0.63 ± 0.06/*p* = 0.000	0.82 ± 0.03/*p* = 0.000	0.78 ± 0.06/*p* = 0.000
STAT3	1	1.03 ± 0.03/*p* = 0.627	1.06 ± 0.07/*p* = 0.376	1.03 ± 0.13/*p* = 0.621	1.08 ± 0.12/*p* = 0.235	1.10 ± 0.05/*p* = 0.135
Cyclin D1	1	0.53 ± 0.08/*p* = 0.000	1.83 ± 0.18/*p* = 0.000	0.57 ± 0.04/*p* = 0.000	0.89 ± 0.07/*p* = 0.174	0.76 ± 0.05/*p* = 0.008

*C:* control, *S:* sorafenib; *F*: 5-FU.

## Discussion

Though few basic scientific studies have provided substantial evidence about the activity of 5-FU in combination with sorafenib in HCC, combined effects of the two agents on other solid tumors are controversial. Thomas and colleagues [16] have shown that single-agent therapy with sorafenib or 5-FU is equally effective in human colorectal cancer, and combination therapy shows no additional effect. On the other hand, a recent study demonstrates that combination therapy of 5-FU and sorafenib exerts a synergistic antitumor effect in renal cell carcinoma [17]. As sorafenib and 5-FU are both commonly used in HCC patients, it is meaningful and instructive to investigate the combined effects in HCC cells.

We find that both sorafenib and 5-FU display antitumor effects in the HCC cell lines MHCC97H and SMMC-7721. Combined effects of the two agents are schedule-dependent: concurrent treatment shows similar efficacy, while pretreatment with sorafenib exacerbates inhibitory effects, but 5-FU pretreatment followed by sorafenib ameliorates inhibitory effects compared with 5-FU monotherapy. According to variations in IC50 values, we find that HCC cells become less sensitive to 5-FU after pretreatment with sorafenib, yet more sensitive when 5-FU pretreatment is followed by sorafenib. That is to say, sequential treatment of 5-FU followed by sorafenib seems to be the optimal schedule for combined administration of the two agents.

Manov and colleagues [18] found that sorafenib, when combined with doxorubicin, increased survival and reduced doxorubicin-induced autophagy by inhibiting MEK/ERK and inducing degradation of cyclin D1 in the HCC cell line Hep3B. Based on these results, they believe that the use of MEK/ERK inhibitors in combination with chemotherapeutics might have possible antagonistic effects. Our results tend to lead to a similar conclusion. Thus, we have tried to understand the mechanism by examining some of the sorafenib-related pathways, like the STAT3 and RAF/MEK/EKR cascade. In addition, we have analyzed cell cycle distribution and expression of proteins associated with cell cycle progression, as it is known that 5-FU is an S-phase-specific chemotherapeutic drug.

Our data reveal that sorafenib efficiently blocks STAT3 and RAF/MEK/EKR pathways, showing down regulation of p-C-RAF, p-ERK, and p-STAT3, while 5-FU shows almost no effect. No changes were observed for total C-RAF, ERK and STAT3 proteins by any of the treatments. Furthermore, sorafenib slows cell cycle progression by inducing a G1-phase arrest, which results in a reduction of the S-phase subpopulation. Sorafenib significantly down regulates cyclin D1 expression in HCC cells, while 5-FU has an opposite effect. Since expression levels of cyclin D1 in combination groups were as well down-regulated, we believe that sorafenib plays a dominant role in regulating cell cycle distributions and cyclin D1 expressions in combined treatments of sorafenib and 5-FU.

Signaling through RAF/MEK/ERK plays a crucial role in cell proliferation, differentiation, malignant transformation, and apoptosis [19,20]. It has been thoroughly demonstrated that sorafenib exhibits remarkable antitumor activity in HCC in vitro and in vivo, through targeting the RAF/MEK/EKR cascade [21,22]. Our results agree well with these reports.

The STAT3 proteins have dual roles as cytoplasmic signaling proteins and nuclear transcription factors that activate a diverse set of genes, including some that are importantly implicated in tumor cell proliferation, survival, invasion, cell-cycle progression, tumor angiogenesis, and tumor cell evasion of the immune system [23-25]. Recently, sorafenib has been shown to suppress tumor growth by decreasing STAT3 phosphorylation in a group of human malignancies [26-29], including HCC [11,30]. As the results we obtained from tests of STAT3 activation after sorafenib treatment are in line with previous studies, we have gained further insight into the mechanism of anti-cancer effects of sorafenib.

It is well known that key genes in cell-cycle control, such as cyclin D1, an important regulator of G1-to-S phase progression [31], are regulated by STAT3 [25,26]. In addition, some studies have demonstrated that cyclin D1 is regulated by both the RAF/ MEK/ ERK and phosphoinositide-3 kinase (PI3K)/Akt pathways [32,33]. Interestingly, some recent studies point out that sorafenib inhibits growth and metastasis of HCC in part by blocking the MEK/ERK/STAT3 and PI3K/Akt/STAT3 signaling pathways [11]; and that sorafenib-induced Tyr705 STAT3 dephosphorylation is mediated by Raf inhibition, as the Raf-inhibitor ZM336372 also results in Tyr705 STAT3 dephosphorylation [34]. Therefore, we have reasons to believe that STAT3 somehow functions downstream of RAF/MEK/ERK signaling.

A recent study has indicated that 5-FU resistance in oral squamous cell carcinoma (OSCC) cell lines HSC-3 and CA9-22, both of which are hypoxia-sensitive (HS), is due to suppressed growth rate and G1-phase accumulation [35]. Similarly, we find that sorafenib causes a G1-phase arrest of HCC cells and, as well, decreases sensitivity to 5-FU, leading to an antagonistic effect of the two agents in the sorafenib-pretreatment strategy.

To summarize, combination effects of sorafenib and 5-FU vary between the different treatment orders. On the whole, antitumor effects are highest in 5-FU pretreatment strategies, and they are lowest following sorafenib pretreatment patterns. Since 5-FU is an S-phase-specific chemotherapeutic drug, it works less efficiently after exposure to sorafenib because of reduction in the proportion of S-phase cells. In contrast, sorafenib exerts further antitumor effects after 5-FU treatments, as the mechanism of sorafenib is cell cycle-independent.

Our in vitro study is limited to the cellular level, and in vivo studies are needed that cover sequential therapy of cell cycle-dependent chemotherapeutic drugs and molecular-targeted drugs. Still, our results do provide some important clues that may help guide drug selection and therapeutic strategy used in clinical treatments.

## Conclusions

From our experimental results and what is known in the literature, we conclude that (1) sorafenib and 5-FU both possess antitumor activity in HCC cells; (2) compared with 5-FU monotherapy, combination treatment with sorafenib and 5-FU shows weaker effects when sorafenib is followed by 5-FU, while the effect is stronger when 5-FU is followed by sorafenib; and (3) sorafenib pretreatment reduces the sensitivity of HCC cells to 5-FU by down regulating cyclin D1 expression via inhibition of RAF/MEK/ERK and STAT3 signaling, which in turn results in G1-phase arrest and S-phase reduction.

## Competing interests

The authors declare that they have no competing interests.

## Authors’ contributions

LD, ZR, QJ, WW, HS, and YW contributed to the study design, analysis, and interpretation of data. YW and ZR conceived the study. LD and QJ performed the experiments. LD and HS participated in statistical analysis. LD drafted the manuscript. ZR and WW carried out the revision and provided important suggestions. All authors read and approved the final manuscript.

## Pre-publication history

The pre-publication history for this paper can be accessed here:

http://www.biomedcentral.com/1471-2407/13/363/prepub
